# Albany County Medical Society

**Published:** 1874-08

**Authors:** F. C. Curtis

**Affiliations:** Secretary


ART. II.—*Albany County Medical Society. Semi-Monthly Meeting held April 22d,* 1874. Reported by F. C. CURTIS, M. D., Secretary.

The President, Dr. SWINBURNE, in the chair.

There were twenty five members present.

After discussing certain business of the Society, a paper was read by Dr. WM. HAILES, on *Cancer of the Liver*, and a case given as follows:

A. B. colored, 75 years old, by occupation a farmer. Came under observation some three or four weeks since. He had always enjoyed good health up to within the last few months of his life, and it was only for the last few weeks that he became at all anxious in regard to the malady under which he was laboring. The amount of inconvenience was so very slight that he was able to be about his accustomed work up to within a month of his death. Upon my first examination of him the following features were noticed: He experienced considerable distress in breathing, and complained of a sensation of smothering about his heart. His abdomen was quite protruberant and a well defined tumor, occupying a position just behind the lower part of the sternum, pressing upon and causing a marked protrusion of the ensiform cartilage. The tumor was firm and nodulated, and pressure upon the growth occasioned intense pain and a spasmodic action of breathing, probably due to embarrassment of the heart's action by direct pressure. The sounds of the heart were quite masked and indicated effusion in the pericardial sac. There was a slight amount of anasarca. His appetite continued good and his bowels regular.

Nothing abnormal noticed in the functions of the stomach.

Diagnosis cancer of left lobe of liver.

Treatment consisted simply in keeping patient comfortable as possible, giving anodynes to allay pain.

The symptoms continued to increase in severity, and his death occurred April 2d, he being under observation about three weeks.

AUTOPSY.

An examination of his abdominal viscera revealed a extensive cancerous disease of the liver, involving both lobes of that organ, enlarging it to a most remarkable degree. It descended a little below the margin of the cartilages of the false rib, but its greatest encroachment was in an upward direction and towards the left side, pressing upon and impeding the action of the heart and lungs. The left lobe of the liver was enlarged and completely filled the hypochondric space. The stomach and spleen were depressed far below their accustomed level. The cancerous growth of the liver had gone on to ulceration and presented a raw and bleeding surface from which had oozed a large quantity of blood, which was found either floating in clots or mixed with the serum in the peritoneal cavity.

A section through the liver revealed numberless nodules of a yellow color projecting from the surface and also occupying deep situations in the liver tissue. The liver weighed 12½ pounds. Further examination revealed a cancerous mass about the size of a small orange involving the coats of the stomach. It was of a firm consistency and situated upon the greater curvature about an inch and a half from the pyloric orifice. There were also numerous enlarged and indurated masses scattered over the surface of the great omentum. The messenteric glands were also considerably involved.

Now, from the facts of the case, what are we to infer in relation to the origin of this malignant growth?

Did it originate in the liver and from this to the other organs, or did it first appear in the stomach and from thence to the surrounding organs and tissues?

We are told that cases of cancer of the liver are of very rare occurrence, and that it is usually the site for secondary growths; that in the majority of cases the stomach is primarily affected, the liver taking on a malignant action secondary to this.

From the existence of so exceedingly extensive cancerous disease and from its being in such an advanced stage, having gone on to ulcerative action giving rise to hæmorrhage, which was the immediate cause of death, and also from the fact that the disease involving the surrounding organs had progressed so very little, it would lead us to infer that it was a case of cancer of the liver proper.

In regard to adjacent organs taking on a cancerous action there are three ways by which this malignancy may infect other tissues.

1st.—By direct influence upon the adjacent structures.

2d.—Through the medium of the lymph.

3d.—Through the medium of the blood.

It would seem, from the examination of this case, that the inference would be that it was by the 2nd method of infection, through the medium of the Lymph, that these tissues became involved, inasmuch that it was confined to a limited area.

Both kidneys contained numerous cysts. The left having one of extraordinary size, it being fully as large as a turkey's egg, besides having a number of smaller ones.

The following case of Cancer of the Stomach and Liver, with circumscribed Peritonitis, was presented by Dr. F. C. CURTIS:

Wm. N. L——d. Act. 49. Occupation, night watchman.

HISTORY.

Patient gives his own history as follows: Early in January—three months ago—he began to notice that he was losing strength. At the same time pallor of the face appeared, and he had some pain in the stomach, not complained of as severe or brought forward prominently. He also vomited his food occasionally. His weakness, or asthenia, was the most remarked. These symptoms came in gradually—increasing, until first seen March 16th. Further questioning elicited the fact that he had had pain in the epigastrium with occasional vomiting for a year back.

He has traveled extensively about the world, in all climates, and was in the army during the war—in the army of the Potomac. He has contracted no diseases from any of these localities, only receiving an injury to his left eye in the army from the limb of a tree, which destroyed the organ. He has always been a strong, healthy man, excepting that several years ago he contracted intermittent fever while living in Greenbush, from which he recovered on moving from there. His family history was not obtained.

March 16th. He came to the office for treatment. As to his condition: his pallor is very marked. He says he is very weak and can hardly keep about, being compelled to sit down frequently. There is some pain or an uncomfortable feeling in the stomach, but the symptoms of disease of this organ are not other than might be referred to a moderately severe case of dyspepsia. Examination about the epigastrium reveals nothing. The liver is of normal size. A constant systolic murmur is heard over the heart—plainest at the base—a roughened blowing sound. The action of the heart is regular. There is no enlargement of the speen, nor are the lymphatic glands enlarged—these all being examined with a view to the possibility of the case being one of leucocythemia. With the same idea the blood was examined microscopically, but no increase of white blood cells was detected, though the examination was not altogether satisfactory.

He came to the office himself for a few times, and then sent word for the first time on the 21st of March that he was unable to leave the house to walk so long a distance. The night previous he was taken with pretty severe vomiting of a brown coffe-colored fluid, which was noted as being much more abundant than the ingesta. Did not complain of suffering much pain, and when seen in the morning was feeling pretty comfortable. For four days he improved, having little pain, though vomiting rather frequently—and although quite weak, was able to walk a few blocks to another house, his family moving at this time. His vomiting was relieved in a measure by a mixture of sweet spirits of nitre, paregoric and soda, and small quantities of fluid food were retained. His countenance was always cheerful.

On the morning of March 25th he reported himself as feeling better than at any time before since the 21st. His weakness was his principal complaint. It was on this morning that a drop of his blood was examined microscopically, his arm being scarified lightly for the purpose, with the negative result already spoken of. He sat in an easy chair by the stove and appeared quite comfortable. In the evening of the same day he was taken with violent vomiting of the coffee-colored fluid, the least attempt to take food of any sort provoking an attack, and even without any exciting cause it came on. After a little, hiccough set in and the two persisted all night in spite of any remedies, leaving him greatly prostrate next morning. There was also severe pain in the epigastrium. He desired cold drinks constantly, though each attempt at taking them brought on fresh vomiting. When seen in the morning he was still having occasional vomiting spells and hiccough, though his strength was very much exhausted. Bismuth and morphia were given dry on the tongue; an occasional pellet of ice, and lime water with milk ice cold in teaspoonful doses were also recommended. The latter, however, could not be retained. A sinapism was applied to the epigastrium. In the evening he was relieved in a measure of the vomiting and pain, and I believe he had very little more hiccough. He was seen at this time by Dr. Swinburne and he was inclined to believe that a little thickening could be detected at the epigastrium.

The other symptoms however left little doubt in the minds of any of the professional gentlemen who saw him of the nature of the malady being other than cancer of the stomach.

The effect of the exhausting night's work and suffering of the 25th, he never rallied from. Although there was not much vomiting after this, yet any attempt at taking food tended to bring it on, and with it increase of pain, and he refused nourishment altogether.

The pain after this was much more marked and constant, and there was some tenderness on pressure. He was a man of remarkable cheerful disposition, and retained this throughout.

There was no febrile action, prostration and pain expressing all his symptoms for the next three days at the end of which he died.

AUTOPSY.

By the express desire of his friends a post mortem examination was made, about 24 hours after death.

In regard to external appearances: There was pallor of the entire body—no waxy or dusky tinge being observable. Emaciation was marked and the muscles very flabby. There was but little rigor mortis. On opening the abdominal walls there was found over the stomach a quantity of thick, yellow pus, and evidence of peritonitis affecting the gastric and diaphragmatic peritoneum. A cancerous mass surrounded the pyloric end of the stomach, extending along the lesser curvaturs for one-third of its length. This appeared broken down on the external surface, a spot of the size of a silver quarter having the look of an ulcer. This was probably the cause of the peritonitis. On the inner surface of the stomach there was still more breaking down of the cancer tissue. The pyloric orifice was open, admitting the passage of the little finger. The stomach was greatly distended, and contained two quarts of coffee colored fluid. This mass of diseased tissue was about the size of the fist and was *completely* covered in front by the left lobe of the liver. The whole mass was very firm. Part of the pancreas was adherent and included in it.

There were also deposits of cancer matter of the size of a walnut and less throughout the liver. These seemed comparatively recent and none of them protruded above the surface of the organ. The liver was not enlarged, weighing 3½ pounds.

One mesenteric gland was enlarged and very hard, being apparently calcaricus. Other abdominal organs were entirely healthy; the spleen quite normal in size and structure. The heart was somewhat enlarged, and there was a patch of atheroma on its surface. The coronary artery was entirely calcified. The aortic valves were not competent and were thickened. A fibrinous clot 16 inches long extended up the aorta and its branches. The lungs were healthy. The brain was examined and found healthy.

Microscopic examination of the deposits in the liver and stomach, by Drs. Van Derveer and Balch, showed them to be those of true cancer.

In this case the diagnosis, though quite certain toward the last, and placed beyond a doubt by the autopsy, was somewhat masked by the absence of one or two almost constant symptoms of the disease.

There was no tumor of the epigastrium. This is said by DaCosta to be the only one symptom at all distinctive of cancer of the stomach. The case is very instructive, not only in the absence of the symptom, but in the cause for its absence being found. The thin left lobe of the liver covered it completely, preventing all posibility of obtaining any evidence of the tumor, which really existed, being obtained by palpation.

Pain is usually another symptom and perhaps as constant as any other, next to tumor. This peculiar pain of cancer—lancinating, piercing, sharp—which we feel ought to be experienced in all cases of cancer growth, is not always present when the deposit is in the stomach walls. In fact, I believe the general verdict is that there is nothing distinctive about it here. It appears not to have been marked in Dr. Haile's case.

It was not augmented by food I believe. As far as the stomach symptoms went there was little to indicate the existence of anything more than chronic indigestion or subacute gastritis, the latter being rendered more probably by the fact of his having been a rather intemperate man. The symptom was not made prominent at all by the patient in giving his history, and only became severe after the setting in of the peritonitis, the occurrence of which is a peculiar feature of the case.

Vomiting was a symptom from the first. This was more characteristic of the disease than either of the others mentioned, especially considering the nature of the matter ejected. Persistent vomiting is suggestive of cancer but not diagnostic. Cases are mentioned of thickening about the pyloric orifice causing it, and so also does *sarcina ventriculi*, pregnancy sometimes, ulcer, &c. But I suppose that in cases of regular vomiting lasting several months, cancer should be first thought of. The coffee ground matter too, is, if thrown off for a long time, characteristic. In gastric ulcer blood is vomited in abundance, if at all, and not in this particular form.

The pallor exhibited in the case is indication of the cancerous cachexia—so to it is of anaemia. There was nothing peculiar about it—nothing of the yellowish tinge spoken of as that of this cachexia.

The marked *weakness*—which was one of the three prominent symptoms from the first—has nothing in it peculiar to cancer.

It was this weakness, with the pallor and the heart murmur, which was at first thought to be anæmie, which led to the thought of lencocythemia. But examination detected no enlargement of the spleen or liver or of the lymphatic glands, and no increase in the number of the white blood-cells was found by the microscope.

Taken together, the pallor, the continued vomiting of coffee colored fluid, and the tired feeling or weakness may be looked on as sufficient upon which to base a diagnosis of cancer of the stomach, even in the absence of any marked pain or of the tumor in the epigastrium, usually spoken of as the most constant symptoms of cancer of the stomach.

The PRESIDENT called attention to the fact that while in this case there was no tumor discernable, there was a more than ordinary communication of the pulsation of the heart to be felt over the epigastrium. This could be caused only by hard tissue, not by a stomach filled with gas or fluid. As to the length of time cancer of the stomach may exist without being fatal, this depended much upon the location of the deposit. It might last for a considerable time without causing serious disturbance if the curvatures are not affected.

Dr. Van Derveer gave a number of cases illustrating various points of the disease, which he has kindly written up for insertion.

Mr. C., married, aged 71, temperate habits, states that for the past 20 years he has had occasional sharp pains in the region of the stomach, and that at times he would vomit his food, yet he considered himself in good health, and could generally attend to his business, that of a carpenter. Saw him first, January 10, 1874. Has been in decided ill health for the past year, failing in flesh and strength, suffering severe pain in the stomach, and most of the time vomiting his food. For the past month his stomach will not retain anything, and only during that time has he been confined strictly to his house. He appears very much emaciated, and extremely pale and cachectic.

In the epigastric region can define a hard tumor the size of a goose egg, painful to the touch, producing, when handled, a sensation of extreme weakness and desire to vomit.

Some œdema of the feet, lungs and other organs appear healthy; urine examined and found normal. Ordered small doses morphine be given with a bitter tonic, also sub-nit. bis. and pepsine.

The latter acted well, but could not bear the tonic and anodyne.

There was no improvement in his case—none was expected—death occurring on January 19, 1874.

Autopsy 24 hours afterwards, decided emaciation. No general anasarca, slight swelling of the feet. Head not examined. Lungs and heart in a healthy condition, very slight pleuritic adhesions, and slight atheromatous change on inner coat of the large arteries.

The coats of the stomach from the cardiac to the pyloric openings were thickened in some places as much as two inches, the deposit scirrhus in character. The cavity of the stomach was almost completely obliterated, and yet the passage through its orifices continued open. It could not hold more than an ounce of fluid. In some places the mass had softened and presented points of ulceration.

The great omentum was filled with hardened masses from a pin head to that of a cherry. Spleen small. Capsule shrunken, adhered decidedly to great end of stomach. Other abdominal organs healthy. Slight effusion of serum in peritoneal cavity.

Scrotum on examination presents double hydrocele, and on a more careful examination each testicle is the seat of a cancerous deposit, the one in the right quite large.

Mr. L., married, aged 62, good habits, always in excellent health, except one severe attack of cystitis ten years ago, from which after a six month's illness made a good recovery. Came to my office December, 1872, presenting a decided case of jaundice, for which he desires treatment. Gave him Pil. Hydrarg Quinine, Dil. Nitro-Mur. Acid and Ext. Tarax alternately until the 12th of January, 1873, without any improvement in his case; is failing in strength and emaciating in flesh. Very little vomiting. Has continued at his work up to the present time. A careful examination reveals decided tenderness over the liver, but cannot make out any tumor in the epigastric region.

April 21st, 1873. To-day, by desire of Mr. L.'s family, am requested to see him with his medical attendant, Dr. H.

He has sought help from many physicians, but his disease has been gradual and certain in its advance. Is very weak, and a mere skeleton in appearance, presents the same deep jaundiced condition as when I last saw him. It is difficult to define the borders of the liver; a hardened mass is felt over the region of the stomach; of late has vomited everything. Urine is found, on examination, loaded with bile, in quantity scant.

Death ensued April 25th, 1873, from starvation.

Autopsy 18 hours after death. Muscles rigid what little is left of them. Encephalon not examined. Organs of Thorax healthy. The pyloric end, and including about one-third of the stomach, was found to be the place of deposit of a cancerous mass as large as a good sized orange flattened. The pyloric orifice of the stomach was closed from the inflammation and ulceration present. The cancerous mass also enclosed a portion of the pancreas, all the vessels going to and from the liver, and a portion of the duodenum. The liver was about half its natural size, and filled with nodules of cancerous growth. Gall bladder shrunken, having in it about a drachm of bile. Spleen very small but healthy. Omentum studded with hardened masses of various sizes. Kidneys healthy. No traces of inflammation about the bladder. Testicles not examined.

Mr. W., aged 65, has suffered for the past fifteen years from hydrocele left side scrotum. Been tapped many many times. For the past year the testicle has become quite hard, much enlarged and paining him constantly. His general condition has been good, but now, from want of sleep and the intense pain he suffers, is failing in strength, and is very desirous of being relieved. Is willing to undergo any operation if he can only get relief from the pain. Has a large scrotal hernia on the same side. In consultation with his attending physician, October 12, 1873, we decided to remove the testicle, which was done. The rupture gave us some trouble, but notwithstanding the patient made a good recovery in three weeks. The testicle is as large as a goose egg; the tunica vaginalis is very much thickened, but the inner part of the mass is found to be on microscopical examination encepaloid in character. The structure is so changed that it is impossible to define any portion of the testicle proper The patient at the present time remains in good health. No symptoms of disease returning.

About second week in April, 1873, W. M. came to the office for treatment; symptom, inability to swallow. On examination with œsophageal tube could detect a decided stricture about four inches down, through which only the smallest size will pass. Looks pale, has lost in flesh, and is weak. Œsophageal bougies were passed twice a week, of various sizes, until at one time I could pass the largest size, with marked relief, up to June 10, 1873, when, on withdrawing the largest one, he complained of some pain, and raised a trifle of blood, while on the instrument there were several pieces of a fleshy-looking substance. This substance under the microscope exhibited small cells, and spindle shaped cells with nnclei. After this did not pass the bougie but two or three times. On telling the old gentleman that we believed his case was one of cancer of the throat, he became very much disheartened, and used to come and almost beg to have us pass the bougie—if only for temporary help, he would say. Was confined to his house for two months previous to his death; not able to swallow much of anything. Suffered much pain, not acute,—more a distress. No family history obtained. His habits had not been good for a greater part of his life. For the past ten years had been a steady drinker of alcohol in some form.

Post-mortem showed ulceration nearly through the anterior and lateral walls of the œsophagus about its middle. The stomach was healthy, containing a small quantity of dark, gumous looking fluid. Microscopical examination showed the diseased tissue to be encephaloid.

August 17, 1873, was called to attend Mrs. A., aged 44, married, mother of four children; usually in good health; had lost the left eye from disease two years ago. Family history good; not well nourished; is over anxious to help provide a home for herself and family, and denies herself much in good diet. Is somewhat jaundiced; bowels constipated; tongue much coated; is failing in strength, yet does her own housework. Menstrual periods regular; feels very much like vomiting at times; urine natural; is losing in flesh; does not sleep well; has much pain in right side. On examination a distinct enlargement of the right lobe of the liver can be felt, extending downwards and inwards towards the umbilicus; is very hard to the touch, and percussion gives acute sickening pain. No disease of any other organ can be detected. Ordered good tonics, nourishment, rest, and sufficient anodyne to relieve pain.

Nov. 1st. Mrs. A. has remained in about the same condition, except that she now occasionally vomits her food. The enlargement has extended down toward the inguinal region and on a level with the navel.

Feb. 1st. Patient has just passed a natural menstrual period, but is now not able to leave her bed; is much emaciated; skin of a dark jaundiced hue; can retain but little food in her stomach; suffers great pain; enlargement much increased, and of a stony hardness and very sensitive; bowels move about once in four days; feces of a light clay color; passes urine quite freely, and in appearance like dark, red wine. A tumor about the size of a butternut can be felt in the left breast, also one much larger surrounding the sternal end of the left clavicle. These tumors feel very hard, and give her much suffering. Anodynes were given freely to quiet pain.

Mrs. A. died March 16, 1874. She did not menstruate after Feb. 1st; is a mere skeleton; all the soft tissues are apparently absorbed; has uot been able to retain anything in her stomach for the past three weeks; bowels not moved during that time. The enlargement of the liver fills almost the entire abdominal cavity. The tumor in left breast has increased in size, while the breast itself seems entirely absorbed; in fact, the tumor and nipple is all that can be defined. The tumor of the clavicle has included the greater portion of that bone. Another tumor of the same stony hardness has developed in the right breast. The dark jaundice of the skin has increased. She appears very much like one suffering from an attack of Addison's disease. The flow of urine has been very scant of late. Mind clear unto death. Though every inducement is offered and every argument advanced, yet the consent of her sisters to hold a post-mortem cannot be obtained.

Case believed to be one of cancer of the liver and *other organs*.

Mrs. C.: Was first called to see her July 29, 1873. Had enjoyed good health up to two years ago last May. Her menstrual periods then ceased, and she noticed soon after that her food after eating gave her some distress. She lost an aunt of some cancerous affection; otherwise her family history is good. To-day she appears pale, thin in flesh, and wears an anxious expression of face. Ten months ago began to vomit her food, also a black coffee-colored looking fluid, in large amount, and for the past two months has been a very troublesome symptom. Bowels constipated. Suffers considerable pain in epigastric and right hypochondriac regions. A distinct tumor can be felt at the lower end, or pyloric orifice, of the stomach. Urine normal. Ordered Sub-Nit. Bis., and to take of anodyne sufficient to allay pain; also injections to move bowels twice a week.

Dec. 1, 1873. Mrs. C. has been more comfortable; does not vomit so much; gets more rest, but is failing in strength and flesh- There is a decided cachexia present; a marked yellowness also of the skin, more prominent at times. Takes very little food. From this time until her death she was kept quiet by use of morphine. There was no further vomiting. At times her bowels would move naturally, and then again the syringe was necessary.

Post-mortem held Jan. 30, 1874. The stomach was found healthy throughout, but three inches below the pyloric orifice, in the duodenum, there was a hard mass, the size of a turkey's egg, which microscopically was found to be cancerous, containing abundant giant cells with nidus and well shown stroma. Rokitanski speaks of cancer of the duodenum as exceedingly rare.

Mrs. V., aged 66, widow. While attending another member of the family my attention was called to her condition, Sept., 1872. Has been confined to the bed for the past two years, and for three years previous had been troubled with dyspepsia, as she had been told. Account of family history not clear, nor the time of her change of life. Is too feeble to give her history. Is a mere mass of skin and bone. For the past two years has vomited much dark-looking fluid and the most of her food. Her daughter states that for the past two months she has absolutely refused to take anything into her stomach, dreading the vomiting and distress that would follow. Is sure that she has not taken an ounce, all told, in that time. A hardened mass can be felt at pyloric end of the stomach. Bowels not moved in two months.

Died Nov. 1, 1872. Post-mortem Nov. 2d. All the organs in the body were found in a healthy condition except the pyloric end and orifice of the stomach, and this, as you see, is closed and surrounded by a hard scirrhus growth not larger than a butternut, and not yet in a state of ulceration, but completely closing the stomach. The lower portion of the rectum was somewhat filled with hardened masses of feces. Bowels had not moved for a period of four months. There had been a steady flow of urine, but at times the bladder would reqnire to be emptied by catheter. No jaundice at any time, but a marked cachexia.

Dr. Bieglow reported an interesting case of peritonitis. He was called upon March 6th to see a lady, aged 58. The history of the case pointed to peritoneal inflammation. She had been complaining of great pain in the right iliac region for a week previous. The abdomen was very tympanitic, and was painful on pressure. All the general symptoms of diffusive inflammation of the peritoneum were present. She died twelve days after the first signs of the disorder manifested themselves.

Post-mortem.—The intestines were distended with gas and adherent by recent deposits of lymph. The duodenum, the lower portion of which was infiltrated with blood, was adherent by recent bands of false membrane to what at first seemed a distended bladder, but which proved to be an ovarian cyst. The pedicle of this was about three inches in length, and twisted upon itself. In twisting a blood vessel had ruptured, and blood flowing out had infiltrated adjacent tissues. This was undoubtedly the cause of the peritonitis. The cyst contained about a quart of bloody-looking fluid, which did not coagulate on standing. Other organs were healthy.

Owing to the lateness of the hour there was no discussion of the case.

